# Temporal trends in mortality related to heart failure and depression in the United States: 1999–2020

**DOI:** 10.21542/gcsp.2025.45

**Published:** 2025-10-31

**Authors:** Pravallika Puvvadi, Akash Ranganatha, Nikita Talpallikar, Emi Kosa Zerzan, Nikhil Vamsi Chitte, Shaheer Ahmad Dar

**Affiliations:** 1 Siddhartha Medical College, Gunadala, Vijayawada, Andhra Pradesh 520008, India; 2 JJM Medical College, Kuvempu Nagar, Davanagere, Karnataka 577004, India; 3 D.Y. Patil Medical College, Laxminarayan Nagar, Kadamwadi, Kolhapur, Maharashtra 416005, India; 4 The University of Texas at Austin, Austin, TX 78712, USA; 5 Kamineni Institute of Medical Sciences, Akkenepally vari lingotam, Narketpalle, Telangana 508254, India; 6 Pakistan Kidney and Liver Institute and Research Center, Murree Road, Adjacent Allama Iqbal Park, near Rawalpindi Cricket Stadium, Rawalpindi, Pakistan

## Abstract

Introduction: Heart failure (HF) remains a leading cause of death in the United States. While depression is known to affect 17–37% of HF patients, its contribution to recorded mortality is poorly characterized in national datasets. Death certificate documentation of comorbid depression in HF deaths provides insight into clinical recognition and reporting patterns rather than the comprehensive burden of disease.

Aims: To analyze temporal trends and demographic disparities in death certificate documentation of depression among decedents with HF as the underlying cause, using the CDC Multiple Causes of Death (MCD) database from 1999–2020.

Methodology: A retrospective observational study was conducted using the CDC MCD database for adults aged ≥25 years in the U.S. from 1999–2020. Deaths with HF (ICD-10: I50) as the underlying cause and depression (ICD-10: F32) as a contributing cause were identified. Age-adjusted mortality rates (AAMRs) per 1,000,000 population were calculated using the 2000 U.S. standard population, and trends were assessed with Joinpoint regression, expressed as annual percentage change (APC) with 95% confidence intervals (CIs).

Results: A total of 3,593 deaths were recorded where HF was listed as the underlying cause and depression as a contributing cause. The overall AAMR for HF with depression showed a declining trend from 1999–2009 (APC = –6.24; 95% CI = –8.31 to –4.12), followed by a plateau from 2009–2020 (APC = 0.05; 95% CI = –0.31 to 0.42). Females accounted for 69.6% of deaths, with higher AAMRs than males. The highest mortality documentation occurred in metropolitan areas and among White individuals (95.1%).

Conclusions: This study reveals declining documentation of depression on HF-related death certificates through 2009, followed by stabilization thereafter. The low absolute count of 3,593 deaths across two decades indicates substantial under-ascertainment compared to the known clinical prevalence of depression in HF patients. These findings primarily reflect documentation and reporting patterns rather than the total mortality burden, emphasizing the need for improved recognition and recording of mental health comorbidities in cardiovascular disease surveillance.

## Introduction

Heart failure (HF) is a clinical syndrome in which structural or functional impairment of ventricular filling or ejection of blood causes signs and symptoms^1^. Around 6.7 million people ≥20 years of age in the United States had HF, and the mortality related to HF in 2022 was 425,000. The American Heart Association projects that the prevalence of HF will increase further, affecting > 8 million adults by 2030, which is 3.0% of the total population. HF prevalence increases with age, and there are racial/ethnic disparities in outcomes among HF patients, for instance, non-Hispanic Black patients experienced a lower rate of death compared with non-Hispanic White patients^2^.

Depression causes a depressed mood or a loss of interest or pleasure in most activities for most of the day, nearly every day. These symptoms impair a person’s ability to perform everyday activities. Depression is one of the most common mental disorders in the United States, 21.0 million adults (8.3% of the adult population) in the United States having at least one major depressive episode. The prevalence of a major depressive episode was highest among individuals aged 18–25, higher among adult females compared to males, and highest among those who report having multiple races^3^. Approximately 1 in 4 adults with HF have depression, Racial/ethnic minorities, younger individuals, and socially vulnerable adults have particularly high rates^4^. However, depression remains significantly underreported on death certificates, often omitted even when clinically diagnosed, leading to substantial under ascertainment of its contribution to mortality statistics**.** This limitation makes national datasets valuable primarily for examining documentation and reporting patterns rather than true disease prevalence.

HF with depression often impairs patients’ self-care ability and is linked to higher cardiovascular and all-cause mortality rates^4^ Dixon et al. ^5^ studied 24,000 Southern Community Cohort Study participants and found that greater frequency of depressive symptoms is associated with higher risk of incident HF. Gathright et al. ^6^ conducted a meta-analysis of 18 prospective studies of depression and mortality in HF, found that depression is related to increased all-cause mortality risk in HF patients. Rollman et al. ^7^ conducted a randomized clinical trial of patients with HF and depression, demonstrating that a blended collaborative care program for treating both HF and depression can improve mental health-related quality of life. While prior studies have explored how these two conditions co-occur and influence clinical trial participants or cohorts from specific communities, few have investigated them in a more heterogeneous, real-world population. This highlights the need to analyze both conditions together in national mortality data in the CDC Multiple Causes of Death (MCD) database.

### Aim and objectives

To evaluate mortality trends in heart failure (I50) where depression (F32) is a contributing cause of death, using national mortality data from the CDC Multiple Causes of Death database from 1999 to 2020. This study stratifies data by sex, race, and geographic location to identify demographic and geographic disparities in mortality patterns.

### Methodology

A retrospective original research study was conducted using the Centers for Disease Control and Prevention Wide-ranging Online Data for Epidemiologic Research (CDC WONDER) Multiple Cause of Death (MCD) database^8^. Data extraction for the study commenced on July 2, 2025. This research involved non–human participants, as the CDC-WONDER database provides de-identified, publicly available data, which ensures that no individually identifiable information is accessible^9^. Therefore, in accordance with regulatory guidance, this study did not require ethics committee approval.

### Case definition and rationale

Deaths among individuals aged ≥25 years from 1999–2020 were included if heart failure (ICD-10: I50) was listed as the underlying cause of death and depression (ICD-10: F32) was recorded as a contributing cause.

Depression was selected as a contributing rather than any mention cause because the “any mention” category includes both underlying and associated conditions, which may overestimate its role in the causal sequence. Restricting analysis to cases where depression was explicitly listed as contributing helps isolate deaths where certifying physicians recognized depression as relevant to the terminal event, allowing for more consistent interpretation of cause-of-death attribution.

Clinically, depression may influence heart failure outcomes through neurohormonal dysregulation, increased inflammation, reduced adherence to therapy, and higher incidence of arrhythmias and rehospitalization. These pathophysiological pathways justify its potential contribution to heart failure–related death and support its inclusion as a contributing cause.

### Coding variability and potential bias

Death certificate coding of psychiatric conditions such as depression is highly variable and often inconsistent across providers, facilities, and jurisdictions. The decision to record depression as a contributing cause is subjective and depends on physician recognition, diagnostic confirmation, and awareness of its relevance to mortality. Consequently, our case definition likely introduces selection bias, preferentially capturing deaths in which depression was both diagnosed and documented, while omitting a large number of cases where it was present but unrecorded. Thus, this dataset reflects documentation and reporting practices, not the total clinical prevalence of comorbid depression in heart failure.

### Data extraction and variables

Data were obtained for all U.S. residents aged ≥25 years with the inclusion criteria described above. The dataset was stratified by:

• Gender: male, female
• Race/Ethnicity: White, Black or African American, American Indian or Alaska Native, and Asian or Pacific Islander
• Urbanization: classified per 2013 NCHS Urban–Rural Scheme into large central metro, large fringe metro, medium metro, small metro, micropolitan, and non-core areas
• Place of Death: medical facility, home, hospice, or nursing home/long-term care facility

Mortality rates were calculated per 1,000,000 of the total U.S. population, not limited to the subset of individuals with heart failure, as national data on the total HF population are unavailable within the CDC MCD framework. The crude mortality rate thus represents population-level frequency rather than disease-specific risk. All rates were age-adjusted to the 2000 U.S. standard population to allow comparability across time and demographic groups.

### Statistical analysis

Descriptive data (counts and percentages) were obtained directly from the MCD database. Temporal trends in age-adjusted mortality rates (AAMR) were evaluated using Joinpoint Regression Program version 5.3.0.0 (National Cancer Institute, November 2024). Annual Percentage Change (APC) and 95% Confidence Intervals (CI) were calculated to assess significant changes in trends over time. Comparisons between HF deaths with versus without documented depression were not feasible because the CDC WONDER MCD interface cannot identify the *absence* of a specific contributing cause across all HF deaths, and under-reporting would bias any proxy denominators. Subgroup APC confidence intervals were reported only where Joinpoint returned stable estimates; series with suppressed or unstable annual rates precluded CI calculation and formal between-group tests.

### Limitations of death certificate data for psychiatric comorbidities

Death certificate data provide insight into how mental health conditions are recognized and recorded rather than their true epidemiologic burden. Psychiatric comorbidities, particularly depression, are often underreported due to diagnostic uncertainty, social stigma, or omission in terminal documentation. Coding practices have also evolved over the two-decade study period, which may influence temporal trends independent of actual mortality changes. Therefore, findings should be interpreted as reflecting patterns in clinical documentation rather than definitive estimates of disease-specific mortality.

## Results

### Demographic characteristics

From 1999 to 2020, the CDC Multiple Causes of Death (MCD) database recorded 3,593 deaths in individuals aged 25 years and older where heart failure (ICD-10: I50) was listed as the underlying cause and depression (ICD-10: F32) as a contributing cause. The crude mortality rate for heart failure with depression was 0.8 per 1,000,000 population, calculated using the total U.S. population as the denominator.

Of the total deaths, males accounted for 1,092 (30.4%) and females for 2,501 (69.6%), indicating a markedly higher mortality burden in women. Regarding race, the majority of deaths occurred among White individuals (*n* = 3,418; 95.1%), followed by Black or African American (*n* = 132; 3.7%), Asian or Pacific Islander (*n* = 30; 0.8%), and American Indian or Alaska Native (*n* = 13; 0.4%) individuals. The predominance of White individuals reflects both population composition and potential disparities in documentation of depression among minority groups.

### Geographic distribution

A majority of deaths occurred in metropolitan areas (*n* = 2,716; 75.6%), while non-metropolitan regions accounted for 877 deaths (24.4%). By place of death, most deaths occurred in nursing homes or long-term-care facilities (*n* = 2,048; 57.1%), followed by home (*n* = 689; 19.2%), medical facilities (*n* = 639; 17.8%), hospice centers (*n* = 44; 1.2%), and other/unspecified locations (*n* = 169; 4.7%). The predominance of nursing-home deaths suggests that depression is more likely to be recorded in settings with longitudinal medical documentation.

### Temporal trends

Overall, the age-adjusted mortality rate (AAMR) for heart failure with depression as a contributing cause showed a significant decline from 1999 to 2009, followed by a plateau through 2020.

• 1999–2006: APC = −4.85 (95% CI −6.1 to −3.5; *p* < 0.05)
• 2006–2009: APC = −18.29 (95% CI −20.4 to −15.9; *p* < 0.05)
• 2009–2020: APC = 0.05 (95% CI −0.4 to 0.5; *p* = 0.72, not significant)

**Figure 1.**
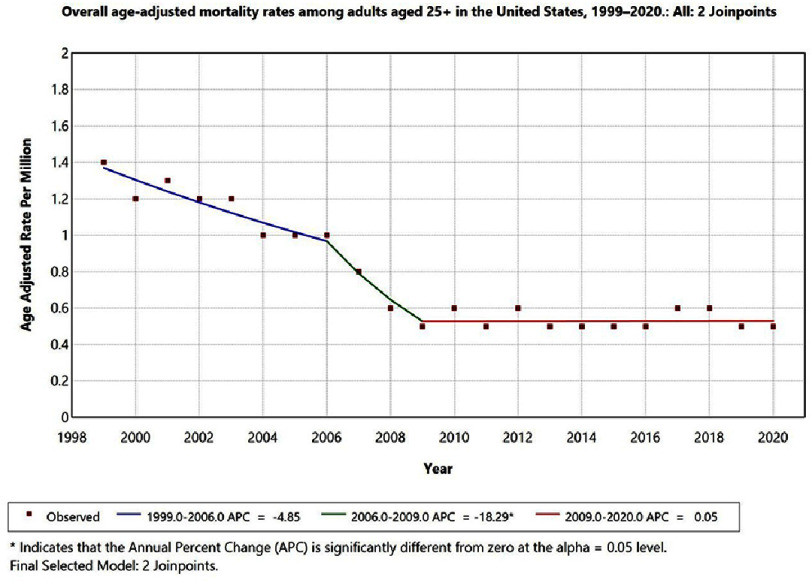
Overall age-adjusted mortality rates among adults aged 25+ in the United States, 1999–2020. The Annual Percentage Change (APC) is significantly different from zero at alpha = 0.05 level.

Significant inflection points were detected in 2006 and 2009 (*p* < 0.05), marking periods of changing documentation and reporting practices. Figure 1 illustrates these changes, with 95% confidence intervals shown as error bands and asterisks indicating statistically significant APCs.

When stratified by gender, females consistently exhibited higher AAMRs than males.

• Females: 1999–2005, APC = −3.33 (95% CI −4.9 to −1.8; *p* < 0.05); 2005–2008, APC = –15.94 (95% CI –17.9 to −13.8; *p* < 0.05); 2008–2020, APC = −1.46 (95% CI −2.3 to −0.5; *p* < 0.05).
• Males: 1999–2006, APC = −5.14 (95% CI −7.2 to −3.1; *p* < 0.05); 2006–2009, APC = –20.73 (95% CI −22.8 to −18.6; *p* < 0.05); 2009–2020, APC = 1.34 (95% CI −0.2 to 2.8; *p* = 0.09, not significant).

**Figure 2.**
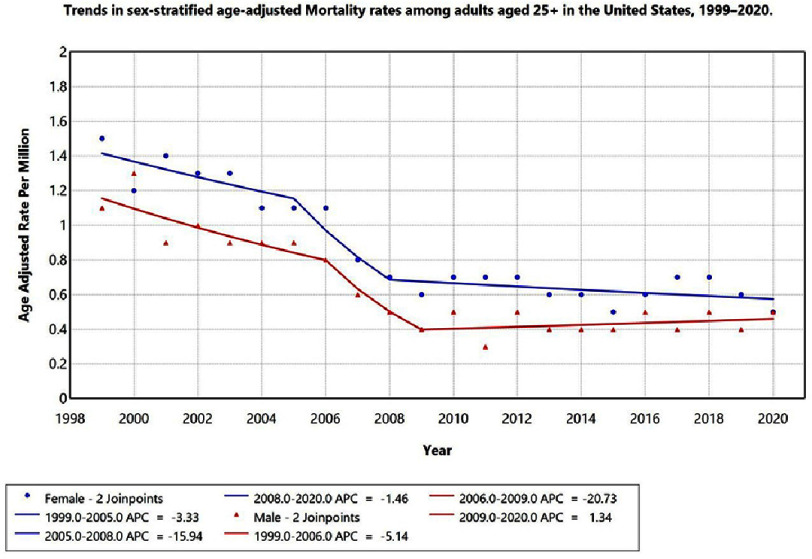
Trends in sex-stratified age-adjusted Mortality rates among adults aged 25+ in the United States, 1999–2020. Anuual Percentage Change (APC) is significantly different from zero at alpha = 0.05 level.

The test for parallelism showed no statistically significant difference between male and female temporal trends (*p* > 0.05), indicating that both sexes followed similar mortality trajectories over time. These results are displayed in Figure 2.

**Figure 3.**
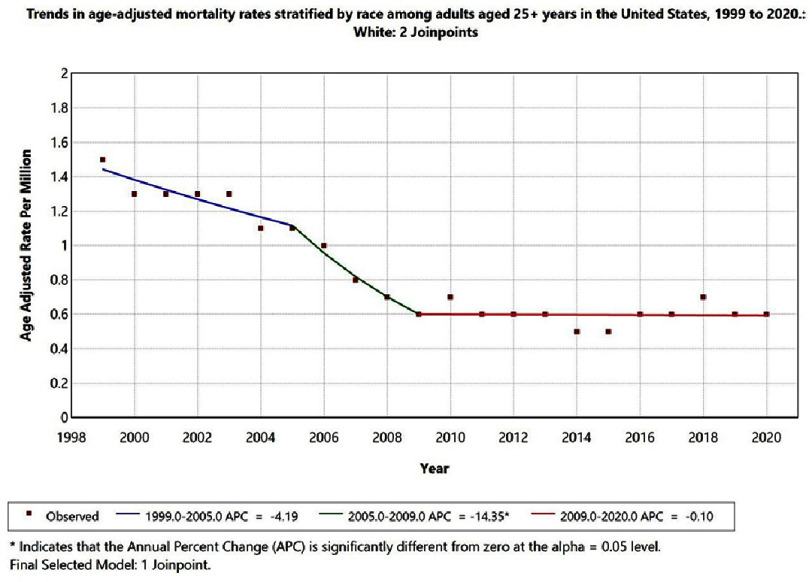
Trends in age-adjusted mortality rates stratified by race among adults aged 25+ years in the United States, 1999 to 2020. Temporal trends for American Indian/Alaska Native, Asian Pacific Islander and Black or African American populations are not displayed due to data suppression for counts < 10, limiting reliable trend analysis.

Racial disparities were less assessable due to data suppression for small counts (<10). Among White individuals, AAMR declined significantly from 1999 to 2009 (APC = −4.19; 95% CI −5.7 to −2.8; *p* < 0.05) and then stabilized from 2009 to 2020 (APC = −0.10; 95% CI −0.6 to 0.4; *p* = 0.68). Data for American Indian/Alaska Native, Asian or Pacific Islander, and Black or African American populations were suppressed in several years, precluding reliable trend estimation. Figure 3 presents race-stratified AAMRs with 95% CIs, with statistically significant APCs marked by an asterisk.

## Discussion

This retrospective study analyzed U.S. national mortality data using the CDC Multiple Causes of Death (MCD) database from 1999 to 2020, where heart failure (HF) was listed as the underlying cause and depression as a contributing cause. A total of 3,593 deaths were recorded in individuals aged 25 years and above. Most deaths occurred in long-term care facilities (57%), followed by homes (19%) and hospitals (18%). The age-adjusted mortality rate declined sharply between 1999 and 2009, then plateaued through 2020. The majority of the patients who died were females (69.6%) and White individuals (95.1%), with most deaths occurring in metropolitan areas (75.6%). Given that clinical studies estimate a 17–37% prevalence of depression among HF patients^11, 12^, the 3,593 deaths identified across two decades likely represent far less than 1% of the expected HF–depression mortality burden. This large discrepancy highlights substantial underreporting of psychiatric comorbidities on death certificates and indicates that these data primarily reflect documentation and coding practices rather than the full epidemiologic burden of HF with depression.

Death-certificate completion practices for psychiatric diagnoses have changed considerably over time. The transition from paper to electronic death registration systems, improved certifier training, and growing mental-health awareness may have increased recognition of depression as a contributing cause of death^8, 9^. Early in the study period, inconsistent reporting likely led to underdocumentation, while the stabilization of rates after 2009 may reflect more uniform use of electronic templates and ICD-10 coding rather than stagnation in clinical outcomes. The apparent plateau in mortality trends therefore likely represents consistency in reporting behavior, not a lack of therapeutic progress. Differences in place of death also influence reporting: nursing homes, which maintain longitudinal problem lists, may more consistently document depression, whereas hospital and hospice deaths often prioritize immediate physiologic causes. Similar underrepresentation of psychiatric comorbidities has been described in other death-certificate analyses, underscoring that such data are best interpreted as reflections of documentation patterns.

Heart failure remains a leading cause of death in the United States, and depression is a well-established comorbidity, affecting 17–37% of hospitalized HF patients^11^. Studies have shown that depression in HF is associated with a 1.5- to 2-fold higher mortality risk compared with non-depressed patients, with one- and five-year mortality rates of 36% versus 33% and 68% versus 63%, respectively^12^. The relationship is bidirectional—depression both contributes to and results from HF, influencing neurohormonal regulation, adherence, and quality of life. Meta-analyses have shown that depression increases all-cause mortality in HF patients by 40% and cardiovascular mortality by nearly 60%^13, 14^. Although our data are consistent with depression being a relevant comorbidity, the very low number of documented cases suggests that these effects are poorly captured in mortality records, reinforcing that national surveillance underestimates the mental-health dimension of HF outcomes.

Women accounted for nearly 70% of HF-depression-related deaths, reflecting the higher prevalence of depression, longer survival with chronic disease, and psychosocial stressors that heighten vulnerability^15^. Men, conversely, may experience worse cardiovascular outcomes because of atypical depressive presentations, lower recognition, and delayed help-seeking^16, 17^. Regarding race, 95.1% of deaths occurred among White individuals, with only 4.9% from minority groups. This likely reflects both population composition and disparities in documentation.

Depression is more often diagnosed and recorded in White patients, while cultural stigma, differential access, and underrecognition contribute to its underreporting among minority populations^16^. Because the CDC WONDER system suppresses annual counts below ten, analyses for smaller racial categories were not possible, limiting our ability to examine true racial or ethnic disparities. Consequently, the stated objective of identifying demographic disparities should be interpreted cautiously or revised in future work, as data availability is insufficient to support detailed subgroup analysis.

Our findings diverge from clinical literature such as Alhassan et al., who reported a stable prevalence of depression (∼26.6%) in HF patients between 2005 and 2020^10^. This discordance likely arises because clinical studies capture diagnosed depression within healthcare systems, whereas death certificates record only conditions explicitly recognized and documented by certifying clinicians. The decline and plateau observed here probably reflect improvements and stabilization in documentation rather than epidemiologic change. Moreover, our dataset encompasses deaths across diverse settings—including hospitals, homes, hospices, and long-term-care facilities—where documentation priorities differ. Many deaths in nursing homes likely represent end-stage disease rather than disparities in care. By contrast, clinical trials such as those by Rollman et al., Chouairi et al., and Angermann et al. enrolled selected patients with detailed phenotyping and follow-up^7, 18, 19^. Thus, our study complements these clinical data by offering a population-level view of how mental-health comorbidities are recorded at death, rather than measured prospectively during care.

Our results are also consistent with broader observations that psychiatric conditions are rarely recorded on death certificates, even when clinically significant^4, 11, 12^. Documentation variability across time, regions, and institutions can obscure true trends, and the apparent improvement seen before 2009 likely reflects enhanced reporting infrastructure rather than actual declines in disease burden. As such, these data are most useful for understanding how recognition and recording of depression have evolved within mortality surveillance systems.

The findings have several implications for practice and policy. Improved training for certifying clinicians and integration of standardized prompts for behavioral conditions in electronic death registration systems could enhance the completeness of psychiatric documentation. More consistent recording would strengthen national mortality surveillance and facilitate research on the mental-health burden of chronic cardiovascular diseases^8, 9^. Future studies linking death-certificate data with clinical and administrative records could validate the accuracy of psychiatric coding and quantify underreporting. Validation efforts comparing death certificates with medical records would be especially valuable for identifying predictors of accurate documentation and guiding quality-improvement initiatives in vital statistics reporting.

This study is subject to several limitations. It relies on death certificate data from the CDC Multiple Causes of Death database, which underascertains the true burden of depression among individuals with heart failure. Depression is often underdiagnosed or omitted from death certificates, so the 3,593 deaths identified likely reflect documentation patterns rather than the full epidemiologic burden. Because of the dataset’s nature, it is impossible to distinguish whether temporal trends represent true mortality changes or shifts in clinical recognition, coding, or reporting practices. The database lacks clinical details such as heart failure subtype, ejection fraction, depression severity, comorbidities, treatment, and socioeconomic variables, limiting control for confounders and precluding causal inference. As a descriptive, ecological analysis, the findings cannot guide clinical decision-making or evaluate therapeutic efficacy. Moreover, the CDC WONDER MCD provides only aggregated data with suppression and no variance estimates, preventing the creation of a valid comparator group without depression, calculation of confidence intervals for all subgroups, or formal statistical testing between demographic categories.

## Conclusions

This national study describes documentation patterns of depression among individuals with heart failure as the underlying cause of death in the United States from 1999–2020. Only 3,593 deaths were recorded, indicating substantial underreporting compared to the known prevalence of depression in HF patients. The trends observed likely reflect changes in documentation and recognition rather than true shifts in mortality.

These findings should be interpreted as reflective of reporting practices, not clinical outcomes. While they underscore the need for better integration of mental health documentation in mortality data, they do not support conclusions about treatment or targeted public health interventions. Future studies linking clinical and behavioral data are needed to more accurately estimate the true burden of comorbid heart failure and depression.
